# Achieving Efficiency of up to 27.01% for SnS-Based Heterojunction Solar Cells Through SCAPS-1D Design and Optimization

**DOI:** 10.3390/nano16211401

**Published:** 2026-11-01

**Authors:** Liping Cai, Jie Huang, Guilin Chen

**Affiliations:** 1 College of Computer and Cyber Security, Fujian Normal University, Fuzhou 350117, China; cailp108@fjnu.edu.cn; 2 Fujian Provincial Engineering Technology Research Center of Solar Energy Conversion and Energy Storage, College of Physics and Energy, Fujian Normal University, Fuzhou 350117, China

**Keywords:** thin-film solar cells, SCAPS, SnS, ZnS:In, conduction band offset, NiO_x_ HTL

## Abstract

Tin sulfide (SnS) has emerged as a highly promising absorber material for thin-film solar cells owing to its excellent optoelectronic characteristics and earth-abundant composition properties. However, it is worth noting that the power conversion efficiency (PCE) of SnS-based thin-film solar cells (TFSCs) is still far below their theoretical potential of approximately 32.0%, and the factors leading to poor performance mainly include high recombination rate, short minority carrier lifetime, and unfavorable conduction band offset (CBO) at pn junction interface. In this study, we selected CdS, ZnSe, and ZnS:In as electron transport layers, NiO_x_, PEDOT: PSS, Spiro-OMeTAD, CuI, and CuSCN as hole transport layers, and compared them through SCAPS-1D simulation to optimize device performance. In addition, the impacts of the SnS absorber layer thickness and doping concentration, bulk defect density, electron transport layer, hole transport layer, and electrode work function on the device performance have been systematically studied using SCAPS-1D. The results show that the solar cell with Al/ITO/ZnS:In/SnS/NiO_x_/Au/SLG architecture can achieve a maximum PCE of 27.01%. This simulation aims to explore the theoretical performance ceiling of SnS-based architecture rather than predicting experimentally realizable efficiency. This study provides theoretical guidance for the design of high-performance, low-cost SnS-based thin-film solar cells.

## 1. Introduction

Thin-film solar cells (TFSCs) have drawn wide interest owing to their uncomplicated manufacturing techniques, minimal material utilization, and remarkable photoelectric conversion efficiency. The IV-VI group compound tin sulfide (SnS) exhibits a bandgap of about 1.35 eV [1,2,3], which is very close to the optimal bandgap of approximately 1.45 eV for the absorber layer in solar cells [4]. Consequently, SnS is considered a highly promising absorber material for sustainable thin-film photovoltaics, owing to its compelling combination of ideal bandgap, high absorption coefficient (>10^4^ cm^−1^), abundance, non-toxicity, cost-effective fabrication potential, and robust stability. Various methods can be employed to prepare SnS thin films, including thermal spray pyrolysis [4], evaporation method [5,6], sol-gel method [7], radio-frequency magnetron sputtering [8], atomic layer deposition [9], chemical bath deposition [10,11], and electron beam evaporation [12]. The p-type SnS thin films obtained via these methods are typically combined with n-type semiconductor thin films to construct heterojunctions, such as CdS [10,13,14], Cd_1−x_Zn_x_S [13], TiO_2_ [15], and SnS_2_ [16].

Previously, numerous researchers have dedicated their efforts to enhancing the photovoltaic performance of SnS TFSCs. In 2006, K.T. Ramakrishna Reddy et al. prepared orthorhombic SnS TFSCs through spray pyrolysis and constructed a p-SnS/n-CdS heterojunction, achieving an efficiency of 1.3% [4]. Subsequently, through optimization of device structure and fabrication techniques, cell efficiency has been progressively improved. For example, the use of MgSnO as a buffer layer [17], pre-purification of SnS powder to suppress secondary phase formation [18], and the preparation of uniform and dense TiO_2_/SnS heterojunctions via spin-coating [19] have increased efficiencies to 2.1%, 3.88%, and 4.8%, respectively. In recent years, the design of multilayer film structures, such as the SnS/Ag/SnS sandwich configuration, has further enhanced short-circuit current density and power conversion efficiency (PCE), reaching up to 5.24% [20].

Alongside experimental advances, simulation studies have provided important theoretical guidance for device optimization. Various modeling works have predicted the efficiency potential of SnS-based solar cells by strategies such as introducing intrinsic layers, optimizing conduction band offsets, adding back surface fields, or employing hole transport layers (HTLs). For instance, Minbashi et al. simulated an efficiency of 7.68% for an ITO/i-ZnO/Zn(O,S):N/SnO_2_/SnS structure [21]. Kumar et al. improved the theoretical efficiency to 8.15% by incorporating an intrinsic layer and a back surface field [22]. The use of Spiro-OMeTAD as a HTL significantly increased the simulated PCE of an ITO/CeO_2_/SnS cell to 25.65% [23]. Furthermore, optimization of heterojunction design, such as adding a P^+^-SnS layer, and comparative studies of different hole transport materials have provided new insights for efficiency enhancement, with simulated efficiencies for some structures approaching 24% [24,25]. In brief, significant progress in the PCE of SnS TFSCs has been achieved through efforts in material preparation, structural design, and simulation-guided optimization. To clearly illustrate this developmental trajectory, Table 1 summarizes the key performance parameters of SnS-based solar cells reported in the literature in recent years.

It is notable that the maximum PCE is 32.6%, predicted by the Shockley–Queisser limit [33,34]. Research shows that the primary factors leading to the suboptimal performance of the device are: (1) The preparation process of the SnS absorber layer tends to induce the formation of secondary phases such as Sn_2_S_3_ and SnS_2_, exacerbating carrier recombination. (2) The unfavorable CBO between SnS and the conventional CdS buffer layer contributes to a weak built-in electric field, resulting in a notably low open-circuit voltage for the device. (3) The increase in series resistance is caused by the back-contact Schottky barrier [25,35,36]. In this study, the impact of different electron transport layers (ETLs), such as CdS, ZnSe, and ZnS:In, on the photovoltaic performance of SnS-based heterojunction solar cells was compared using the one-dimensional Solar Cell Capacitance Simulator (SCAPS-1D) software. Various HTLs, including NiO_x_, PEDOT:PSS, Spiro-OMeTAD, CuI, and CuSCN, were also selected to enhance the output performance of the heterojunction TFSCs. The efficiency of the SnS-based solar cells was improved by varying material parameters, such as the thickness of each layer, doping concentration, defect density, and metal work function.

## 2. Simulation and Methods

### 2.1. Numerical Modeling

We utilized SCAPS-1D (version 3.3.7) for our simulations. The software enables the construction of target solar cell structures based on the parameters of the studied cell type and the setting of various necessary key parameters. It generates numerical results for the model by solving key semiconductor equations (including Poisson’s equation and the continuity equations for electrons and holes), and acquires data such as the quasi-Fermi levels of charge carriers, the current density–voltage (*J*–*V*) characteristic curves of solar cells, and electric field distribution through numerical calculation techniques [37,38]. The three equations are respectively presented as follows:

(1) $$\frac{d^{2}}{dx^{2}}\psi (x)=\frac{e}{\varepsilon_{0}\varepsilon_{r}}(p(x)-n(x)+N_{D}-N_{A}+\rho_{P}-\rho_{N})$$

(2) $$\frac{dJ_{n}(x)}{\mathrm{dx}}-e\frac{\partial n(x)}{\partial t}-e\frac{\partial \rho_{N}}{\partial t}=G(x)-R(x)$$

(3) $$\frac{dJ_{p}(x)}{\mathrm{dx}}+e\frac{\partial p(x)}{\partial t}+e\frac{\partial \rho_{N}}{\partial t}=G(x)-R(x)$$

Here, the symbol *ψ* stands for the electrostatic potential, while *e* signifies the fundamental charge carried by an electron. The term *ε*_0_ is the vacuum permittivity, and *ε_r_* denotes the relative permittivity of the material. The variables *p* and *n* correspond to the concentrations of holes and free electrons, respectively. Additionally, *N*_A_ indicates the concentration of acceptor impurities, whereas *N*_D_ refers to the concentration of donor impurities. The functions *ρ_N_* and *ρ_P_* describe the spatial distribution of free electrons and holes, respectively. Meanwhile, *J_n_* and *J_p_* represent the current densities associated with free electrons and holes, respectively. Finally, *G*(*x*) and *R*(*x*) denote the charge generation rate and recombination rate [37].

### 2.2. Structure and Simulation Parameters

**Figure 1:**
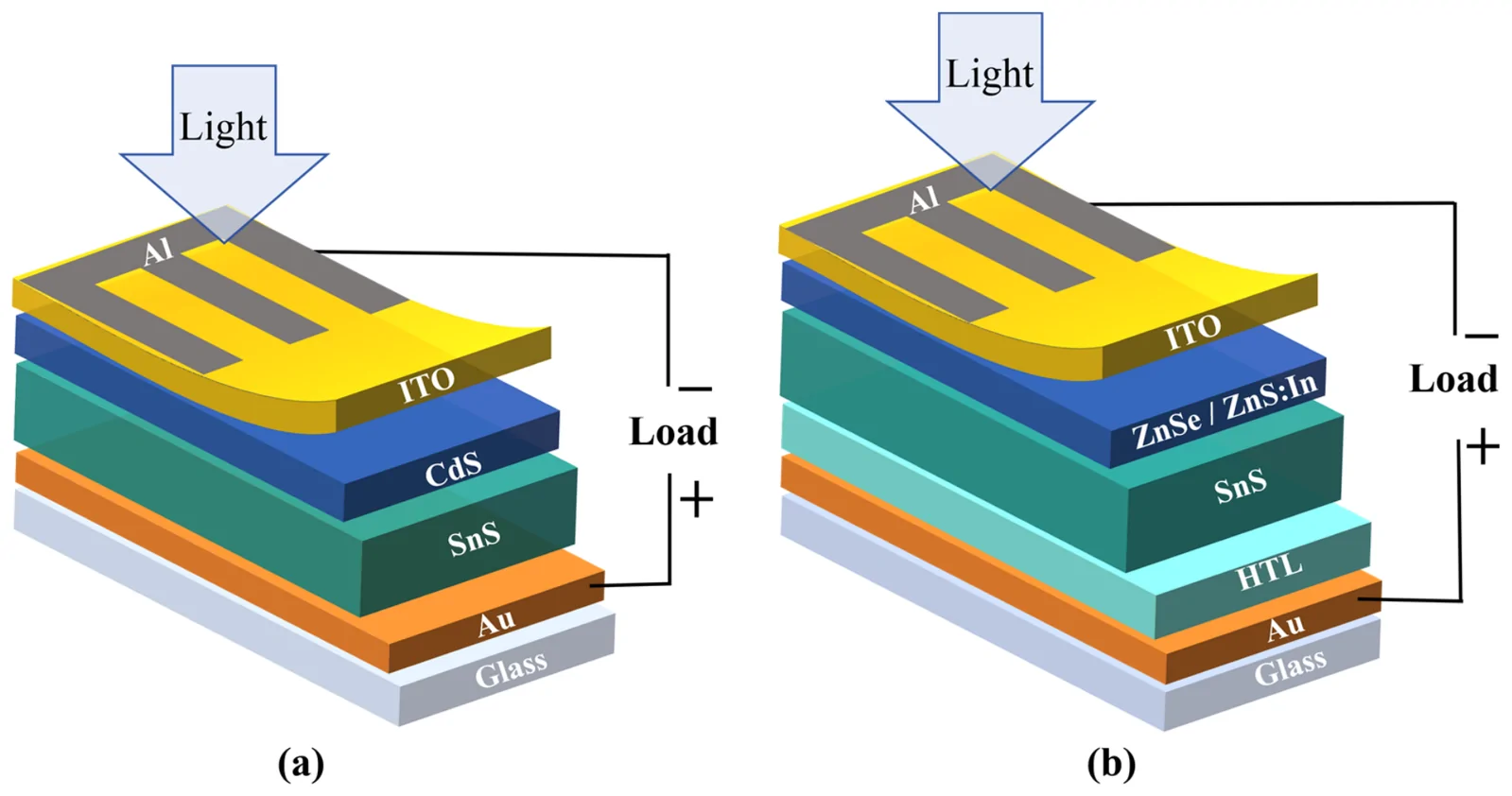
Schematic structure of the solar cells. (**a**) Al/ITO/CdS/SnS/Au/SLG. (**b**) Al/ITO/(ZnSe, ZnS:In)/SnS/HTL/Au/SLG.

The main configuration of SnS-based solar cells consists of a sequence of thin layers, including a front contact layer, buffer layer, absorber layer, and back-contact layer, as shown in Figure 1. Figure 1a depicts the schematic structure of Al/ITO/CdS/SnS/Au/SLG, with SnS as the p-type absorber layer, CdS as the n-type semiconductor buffer layer, ITO as the window layer, and Au as the back-contact material. Figure 1b presents the redesigned SnS-based TFSC architecture with the configuration Al/ITO/(ZnSe, ZnS:In)/SnS/HTL/Au/SLG. In this configuration, ZnSe and ZnS:In serve as n-type semiconductor buffer layers, replacing the conventional CdS layer. Additionally, a HTL is introduced between the absorber layer and the back contact to facilitate hole transport, block electron transmission, increase interfacial contact, and make energy level alignment easier, which increases the PCE of the cells [39].

To address the limitations of the conventional CdS buffer layer in SnS-based solar cells, we proposed ZnSe and ZnS:In as alternative ETL materials. The conventional CdS buffer layer is environmentally undesirable because it contains toxic cadmium. In addition, compared to CdS with a bandgap of 2.4 eV, ZnSe and ZnS:In (In%: 0–12%) possess wider bandgaps. This reduces the absorption of short-wavelength photons, allowing more photons to penetrate the SnS absorber layer, thereby enhancing the light utilization efficiency [40,41].

To simulate and analyze the output performance of the proposed TFSCs using the SCAPS-1D software, it is essential to set the physical parameters for each layer. The parameter settings for each layer material and interfaces are provided in Table 2 and Table 3, with values sourced from reliable experimental and literature reports [25,37,38,39,40,41,42,43,44,45,46]. The 80 nm buffer layer thickness listed in Table 2 is the initial baseline parameter of the buffer layer before thickness optimization. The baseline value refers to existing literature (references [44,45,46]), and the conventional thickness range for this type of buffer layer is 50–100 nm. We selected 80 nm as the uniform intermediate value to fairly compare the device performance of three different buffer layers, CdS, ZnSe and ZnS:In, under the same baseline conditions. Under typical test conditions, the simulations were carried out with an AM 1.5 spectrum and an intensity of 1000 W/m^2^ for front-side illumination while keeping the temperature at 300 K in the surrounding area. The absorption coefficient of SnS was adjusted at 10^5^ cm^−1^. The approximate thermal velocities of both electrons and holes in all layers were uniformly assigned as 10^7^ cm/s. For Al and Au contacts, both the electron and hole surface recombination velocities were established at 10^7^ cm/s. It was assumed that defects followed a neutral Gaussian distribution with a characteristic energy of 0.1 eV.

## 3. Results and Discussion

In this work, a sequential single-parameter optimization strategy is adopted. Within each parametric study subsection, only one physical parameter is varied while all other parameters are kept fixed at the baseline values. These parametric sweeps correspond to intermediate device configurations and are used to identify the individual contribution of each parameter to photovoltaic performance. After completing all single-parameter investigations, we integrate the optimal values obtained from each sweep and construct a unified final device structure. All final *J–V* characteristics, quantum efficiency curves, and the reported photovoltaic parameters (PCE = 27.01%) are simulated using this single consistent device configuration. It should be noted that sequential single-parameter optimization cannot fully explore the entire multi-dimensional parameter space, but this approach is widely used in SCAPS-1D simulations to decouple the independent effect of each physical variable.

### 3.1. Effect of CBO and VBO at the Absorber/Buffer Boundary

In accordance with Anderson’s rule, the vacuum levels of two distinct semiconductors at a heterojunction interface are required to maintain continuity in energy band diagrams. Such discontinuities between neighboring semiconductor energy bands are referred to as band offsets in heterojunction systems. The offset originates from the difference between the bandgap energies of the constituent materials, primarily including the conduction band offset (CBO) and the valence band offset (VBO). The CBO at the absorber-buffer interface is crucial as it determines the heterojunction device’s carrier transport and recombination characteristics [47,48]. The interfacial CBO manifests as either positive or negative polarity configurations, which are theoretically governed by Equations (4) and (5) [48]. TL in the formula refers to ETL and HTL.

(4) $$\mathrm{CBO}=\Delta E_{c}=E_{c,\mathrm{SnS}}-E_{c,\mathrm{TL}}=\chi_{\mathrm{TL}}-\chi_{\mathrm{SnS}}$$

*VBO* = Δ*E_v_* = *E_v_*_,*SnS*_ − *E_v_*_,*TL*_ = (*χ_TL_* + *E_g_*_,*TL*_) − (*χ_SnS_* + *E_g_*_,*SnS*_) (5)

**Figure 2:**
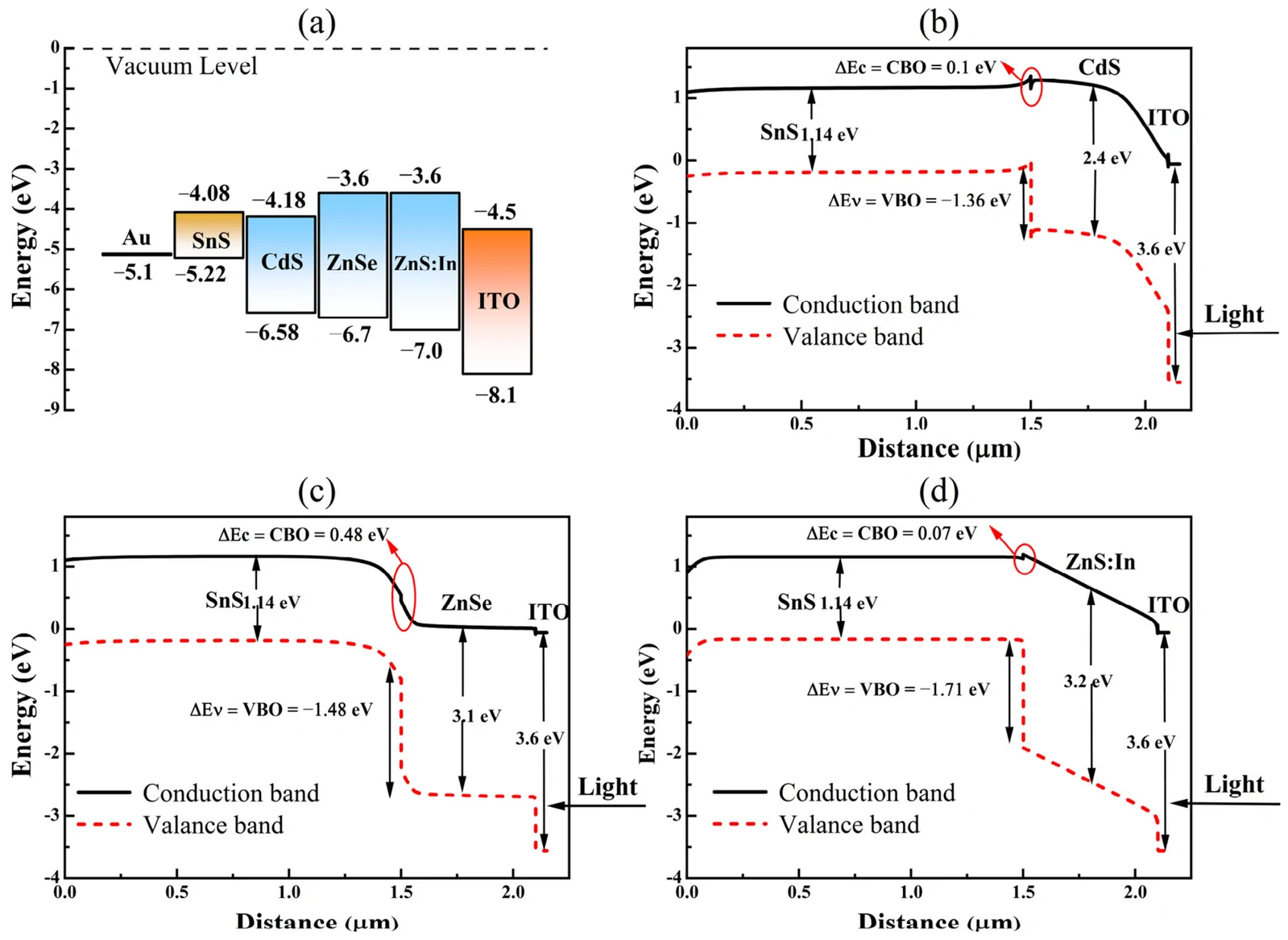
The ITO/CdS (ZnSe, ZnS:In)/SnS/Au energy band structure. (**a**) Energy band diagram. (**b**) CdS. (**c**) ZnSe. (**d**) ZnS:In.

Figure 2 displays the ITO/CdS (ZnSe, ZnS:In)/SnS/Au energy band structure. As shown in Figure 2b, the buffer layer CdS exhibits good valence band alignment with the absorption layer SnS, while forming a “spike-like” (0.1 eV) profile in the conduction band [36]. However, considering the toxicity and environmental unfriendliness of Cd, alternative buffer layers need to be investigated. The energy band structure of semiconductors, including the positions of conduction and valence bands, is a critical factor determining their optoelectronic properties. Through compositional adjustment or doping, the band structures of semiconductors can be effectively engineered. Figure 2c reveals a conduction band offset of 0.48 eV between ZnSe and SnS, which is a mild “spike-like” alignment. Figure 2d shows that the absorption layer SnS has a higher conduction band than the window layer ZnS:In, forming a slight “spike-like” band alignment. Research indicates that both bandgap energy and electron affinity (*χ*) of indium (In) -doped ZnS thin films vary with dopant concentration, decreasing from 3.53 eV (In%: 0%) to 2.94 eV (In%: 12%) [49]. The optimized value for the bandgap was found to be approximately 3.1 eV, at which point the CBO is about 0.07 eV, as determined through iterative simulations. This small offset facilitates easy electron transport from SnS to ITO through the ZnS:In layer, while the significant valence band offset effectively blocks hole flow toward the ITO layer, thereby reducing carrier recombination [33,50]. Additionally, ZnS:In avoids the toxicity issue of CdS. Consequently, ZnS:In is a promising replacement for the CdS buffer layer.

**Figure 3:**
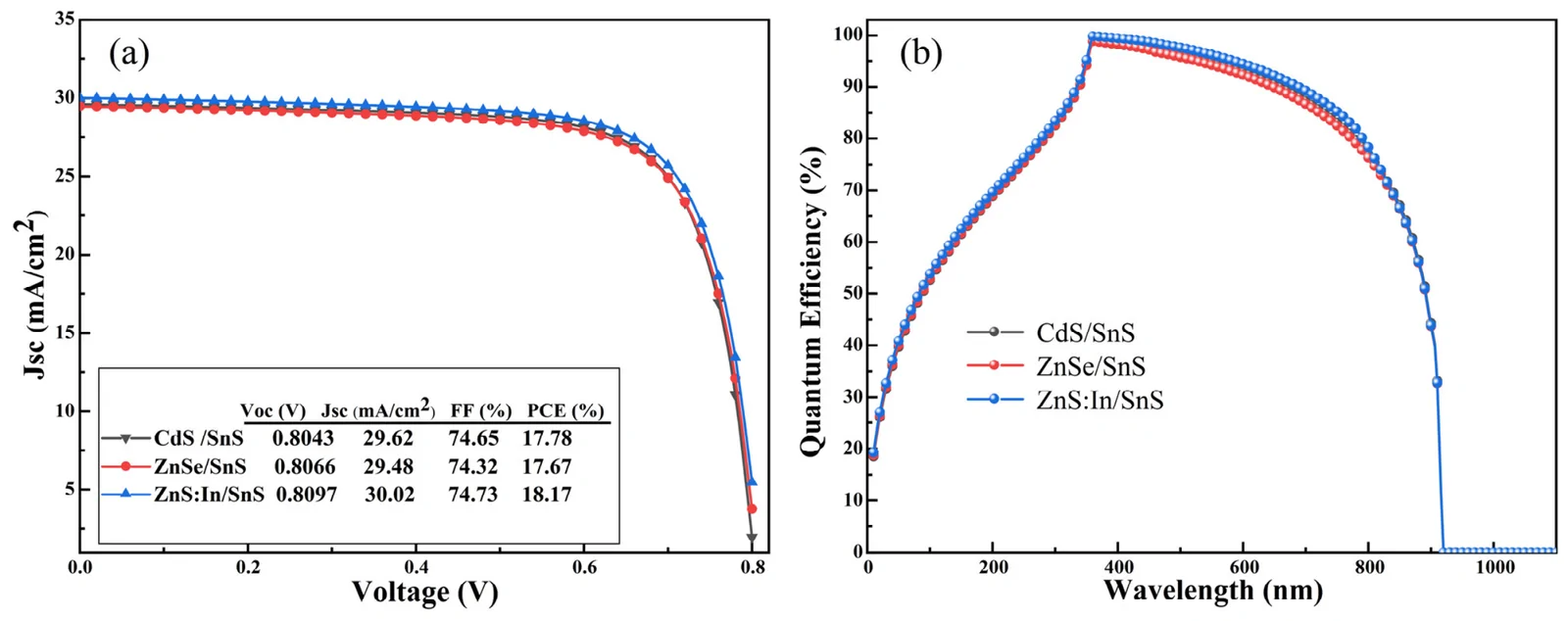
Comparison of photovoltaic parameters with different buffer layers. (**a**) *J-V* characteristic curve. (**b**) The external quantum efficiency.

As analyzed above, the CBO and energy band alignment at the ETL/SnS interface directly determine whether ETLs can effectively fulfill their core functions. The ETL significantly enhances the PCE by facilitating charge separation and suppressing recombination. The choice of ETL materials is a crucial research area for optimizing devices [40,51]. This study simulates the performance of CdS, ZnSe, and ZnS:In as ETLs, with their parameters outlined in Table 2, along with the associated *J–V* curves and quantum efficiency (QE) spectra displayed in Figure 3a and Figure 3b, respectively. As shown in Figure 3a, the *J–V* curves of ZnSe and CdS nearly overlap, while ZnS:In exhibits comparatively superior performance, achieving a FF of 74.73% and a PCE of 18.17%. This is attributed to the favorable band alignment and lower contact barrier at the interface between ZnS:In and the absorber layer [36,47]. Figure 3b reveals that the QE spectra of these materials all exceed 90% in the short-wavelength range (350–600 nm), indicating high utilization efficiency of incident light. Notably, the superior band alignment enables more balanced carrier injection and suppressed interface recombination, thereby accounting for its generally higher QE performance over ZnSe and CdS. In contrast, ZnSe and CdS exhibit certain limitations in their QE performance: the former is mainly constrained by a larger conduction band offset, while the latter suffers from relatively higher interface recombination risk. In conclusion, ZnS:In can serve as an ideal electron transport layer material for SnS-based solar cells.

### 3.2. Impact of SnS Thickness and ZnS:In Thickness on Device Performance

The thickness of the absorption layer and buffer layer in solar cells are two key parameters, and the thickness setting has a significant impact on the performance parameters. To investigate how the thicknesses of the absorption layer and buffer layer affect their photovoltaic characteristics, ZnS:In was chosen as the electron transport layer. We adjusted the thickness of the SnS absorption layer in 8 steps, ranging from 0.5 µm to 2 µm. Meanwhile, the thickness of the ZnS:In buffer layer varied in 10 steps, ranging from 0.01 µm to 0.1 µm. During the whole operation process, in accordance with the requirements listed in Table 2, all other parameters were kept constant.

**Figure 4:**
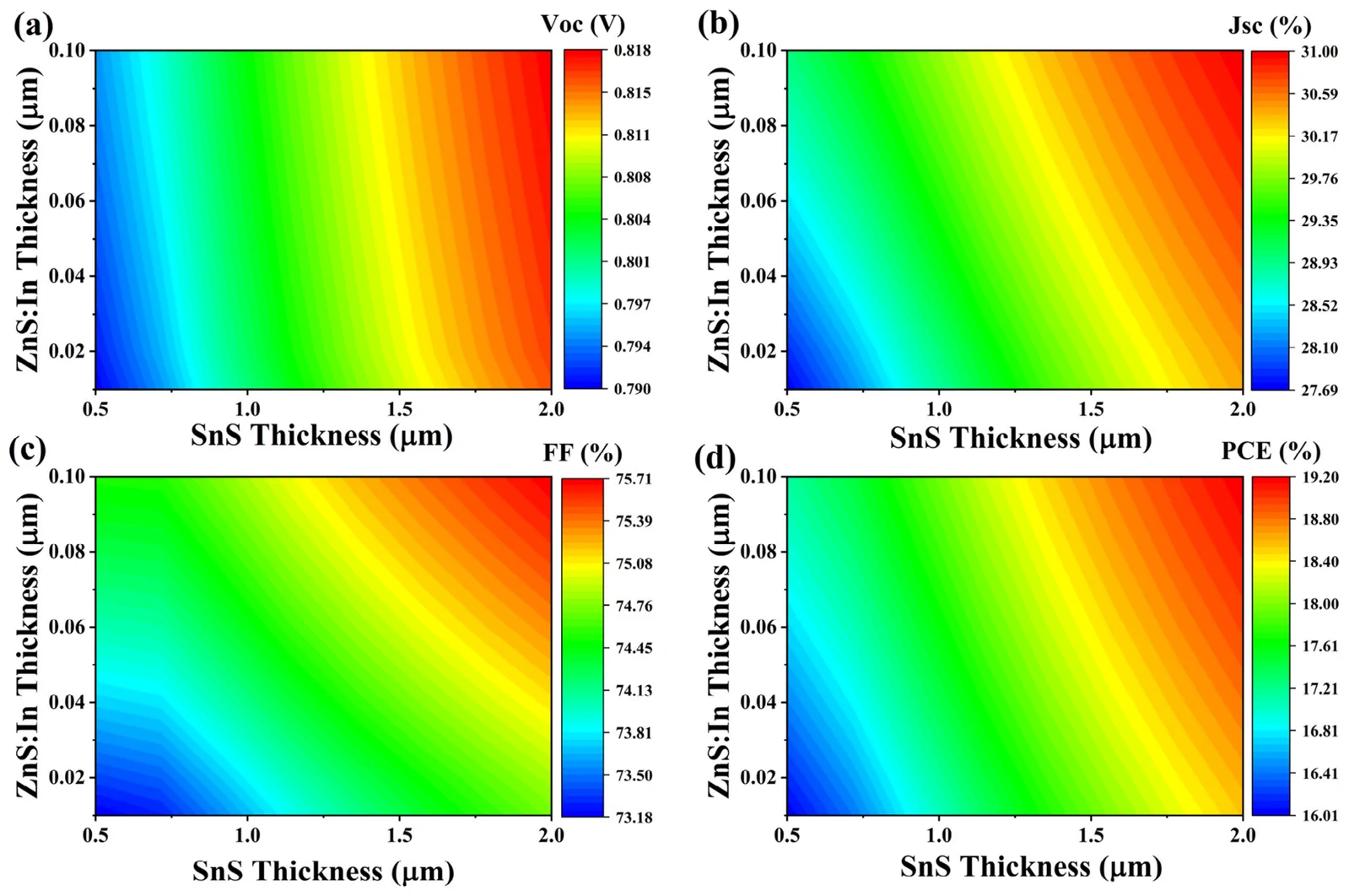
Contour plots of the variation of the photovoltaic performance with the thickness of the SnS and the thickness of the ZnS:In. (**a**) *V*_OC_. (**b**) *J*_SC_. (**c**) FF. (**d**) PCE.

Figure 4 illustrates the influence of the absorption layer and electron transport layer thicknesses on the photovoltaic performance of the solar cell. As shown in Figure 4a, the *V*_OC_ stabilizes around 0.810 V when the SnS thickness ranges from 1.5 µm to 2 µm, with variations in ZnS:In thickness showing no significant impact on *V*_OC_. Figure 4c indicates that the FF remains consistently above 75.02% for SnS thicknesses of 1.5–2.0 µm and ZnS:In thicknesses of 0.06–0.1 µm. Figure 4b and Figure 4d demonstrate that the *J*_SC_ and PCE reach the values of 30.5 mA/cm^2^ and 18.64%, respectively, when the SnS thickness is 1.5 µm and the ZnS:In thickness is 0.08 µm. For the ultrathin ZnS:In buffer layer, insufficient thickness (below 0.04 µm) may cause incomplete surface coverage and introduce shunting leakage pathways. As the ZnS:In thickness increases toward 0.08 µm, sufficient heterojunction passivation is realized without inducing severe parasitic optical absorption or extra series resistance. Further increase of ZnS:In thickness above 0.08 µm leads to slight parasitic short- wavelength photon absorption inside the buffer layer and elevated series resistance, both of which degrade device performance [51,52,53]. The ZnS:In/SnS heterojunction solar cell achieves a peak PCE approaching 19% when the SnS absorption layer thickness is between 1.5–2.0 µm and the ZnS:In electron transport layer thickness is in the range of 0.06–0.1 µm. Considering the experimental fabrication feasibility for obtaining continuous, pinhole-free ultrathin ZnS:In films, we fixed the optimized parameters as SnS (1.5 µm) and ZnS:In (0.08 µm) for all subsequent simulations and discussions.

### 3.3. Impact of the Doping Concentration on Absorber and Buffer Layers

The doping concentration of acceptors and donors in the absorption layer and buffer layer is also one of the key factors affecting the performance of solar cells [54]. In order to find the optimal concentrations of acceptor carriers and donor carriers, we conducted numerical simulations to investigate the effects of acceptor concentration in the absorption layer and donor concentration in the buffer layer on the photovoltaic performance of ITO/ZnS:In/SnS/Au solar cell structures. During the simulation process, the optimized thicknesses of 1.5 µm and 0.08 μm were chosen for the absorption layer and buffer layer. The acceptor concentration in the SnS layer varies from 10^13^ to 10^18^ cm^−3^, while the donor concentration in the ZnS:In layer ranges from 10^15^ to 10^20^ cm^−3^, while maintaining a fixed doping concentration of 1 × 10^21^ cm^−3^ in the ITO window layer.

**Figure 5:**
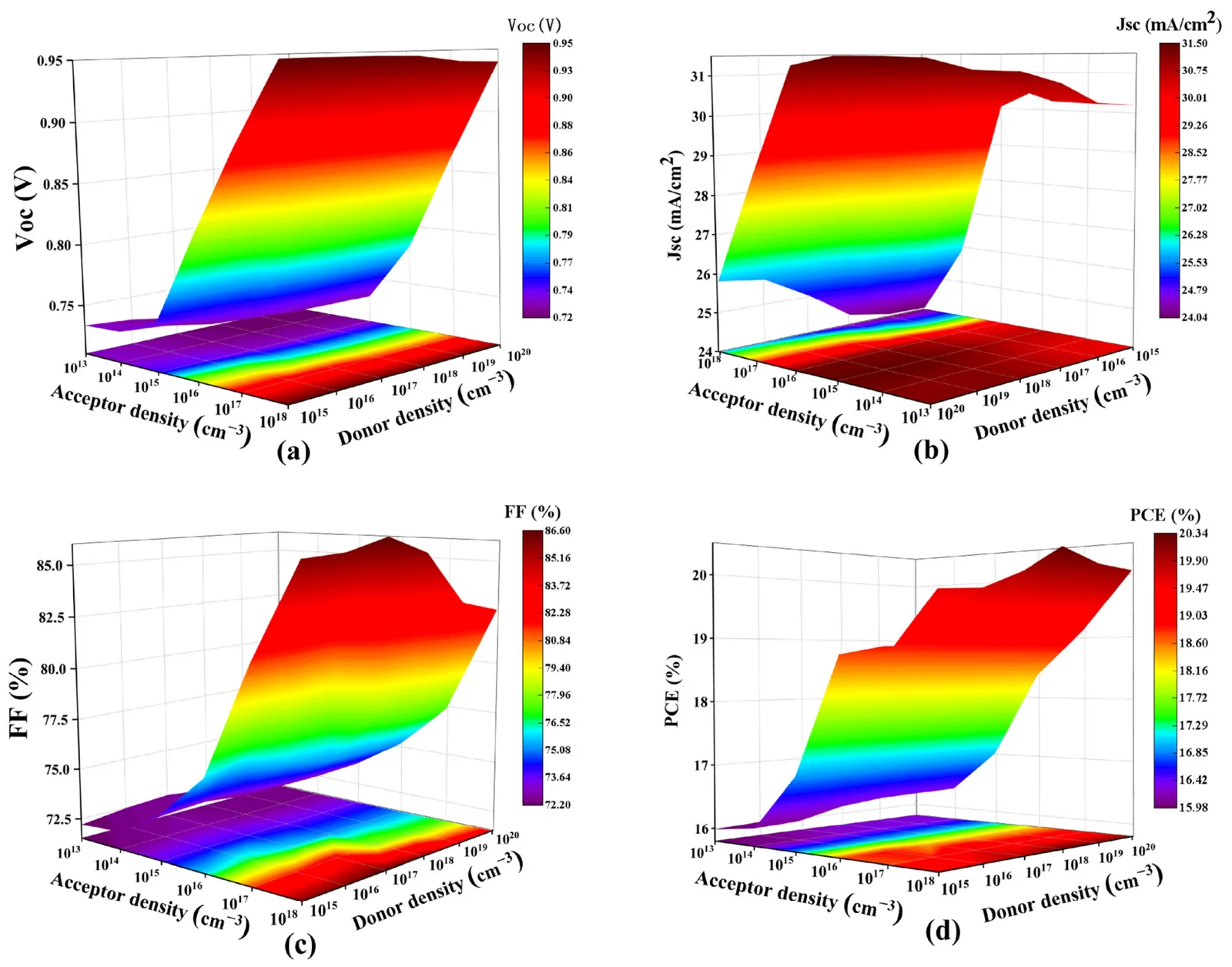
The influence of acceptor density and donor density on photovoltaic parameters. (**a**) *V*_OC_. (**b**) *J*_SC_. (**c**) FF. (**d**) PCE.

Figure 5 shows the influence of acceptor density and donor density on photovoltaic parameters. The acceptor density of the absorber layer was increased from 10^13^ to 10^18^ cm^−3^, resulting in an increase in the *V*_OC_, FF, and PCE from 0.91 V to 1.14 V, 79.07% to 89.12%, and 18.94% to 26.22%, respectively, but a decrease in *J*_SC_ from 31.49 to 28.85 mA/cm^2^. When the density of acceptor carriers was above 10^17^ cm^−3^, the maximum efficiency of the proposed solar cell can be obtained. However, within this optimal acceptor concentration range, variations in donor carrier density show negligible influence on the photovoltaic performance parameters.

**Figure 6:**
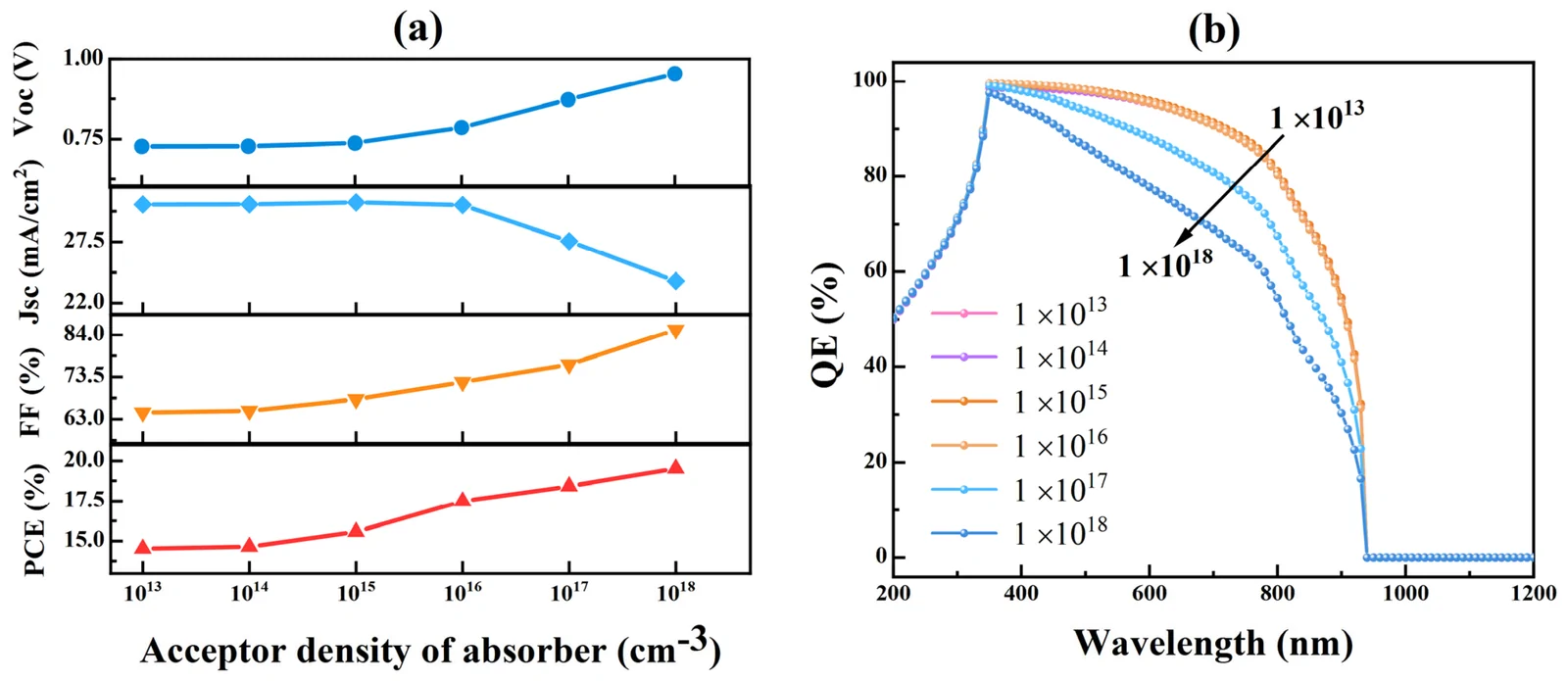
Effect of the performance parameters of the solar cell with different acceptor doping density. (**a**) Absorber doping density. (**b**) QE spectrum.

Figure 6a demonstrates the changes in *V*_OC_, *J*_SC_, FF, and PCE as a result of varying acceptor and donor doping levels. As we know, the built-in potential (*V_bi_*) inside the solar cell is given by Equation (6) as follows [55]:

(6) $$V_{\mathrm{bi}}=V_{T}\ln\frac{N_{A}\times N_{D}}{n_{i}^{2}}$$

The built-in potential is represented by *V_bi_*, the thermal voltage is shown by *V_T_*, the intrinsic carrier concentration is denoted by *n_i_*, and the acceptor and donor doping concentrations are represented by *N_A_* and *N*_D_, respectively. As shown in Figure 6a, the photovoltaic parameters of the solar cell generally exhibit an upward trend with the increase of *N_A_* in the SnS layer, which is consistent with the report in Reference [56]. The increase in *N_A_* enhances both the *V_bi_* and the strength of the built-in electric field, thereby improving the *V*_OC_ and promoting carrier separation, which consequently leads to an enhancement in FF. However, high doping simultaneously results in a significant narrowing of the space charge region (SCR) width [57]. Within the long-wavelength range of 600–900 nm, the combination of greater photon absorption depth and the narrowed SCR causes a large number of photons to be absorbed in field-free regions. Meanwhile, the high doping level itself intensifies the recombination rate. Together, these effects lead to the decrease in QE shown in Figure 6b (e.g., the QE at 800 nm drops from 80.66% to 54.46%). Therefore, the optimal acceptor doping concentration for p-SnS is determined to be 10^18^ cm^−3^.

**Figure 7:**
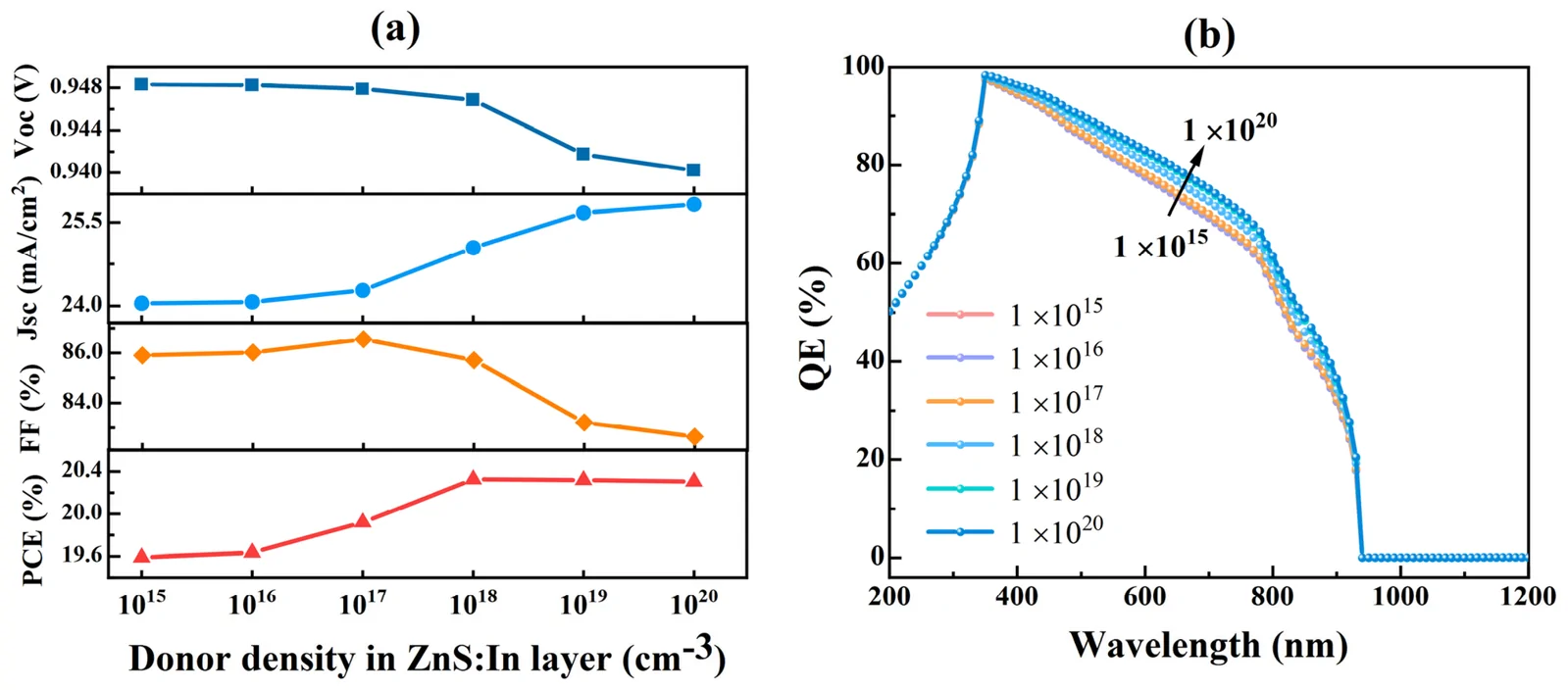
Effect of the performance parameters of the solar cell with different donor doping density. (**a**) Donor doping density. (**b**) QE spectrum.

Figure 7a demonstrates that the device achieves optimal performance with a ZnS:In doping concentration above 10^18^ cm^−3^, resulting in a PCE of 20.33%, FF of 85.71%, *J*_SC_ of 25.05 mA/cm^2^, and *V*_OC_ of 0.947 V, after which the performance slightly declines. Moderate doping increases the electron concentration in ZnS:In, which reduces the bulk resistance and contact resistance of the buffer layer, thereby facilitating electron transport and improving *J*_SC_ [58]. However, when the doping concentration exceeds 10^20^ cm^−3^, defect states promoting non-radiative recombination are introduced, leading to degraded device performance. As can be seen from Figure 7b, when the doping concentration of the ZnS:In buffer layer increases from 10^15^ cm^−3^ to 10^20^ cm^−3^, the QE in the 400–900 nm wavelength range shows a slight improvement. This enhancement is attributed to the moderate doping, which improves the conductivity of the buffer layer and promotes the formation of an impurity energy band, thereby significantly enhancing carrier collection efficiency and broadening the light absorption range, ultimately leading to the observed increase in QE [58]. These observations highlight the crucial role of doping-modulated recombination dynamics in determining the wavelength-dependent optoelectronic performance of the device.

### 3.4. Effect of Defect Density on Device Efficiency

**Figure 8:**
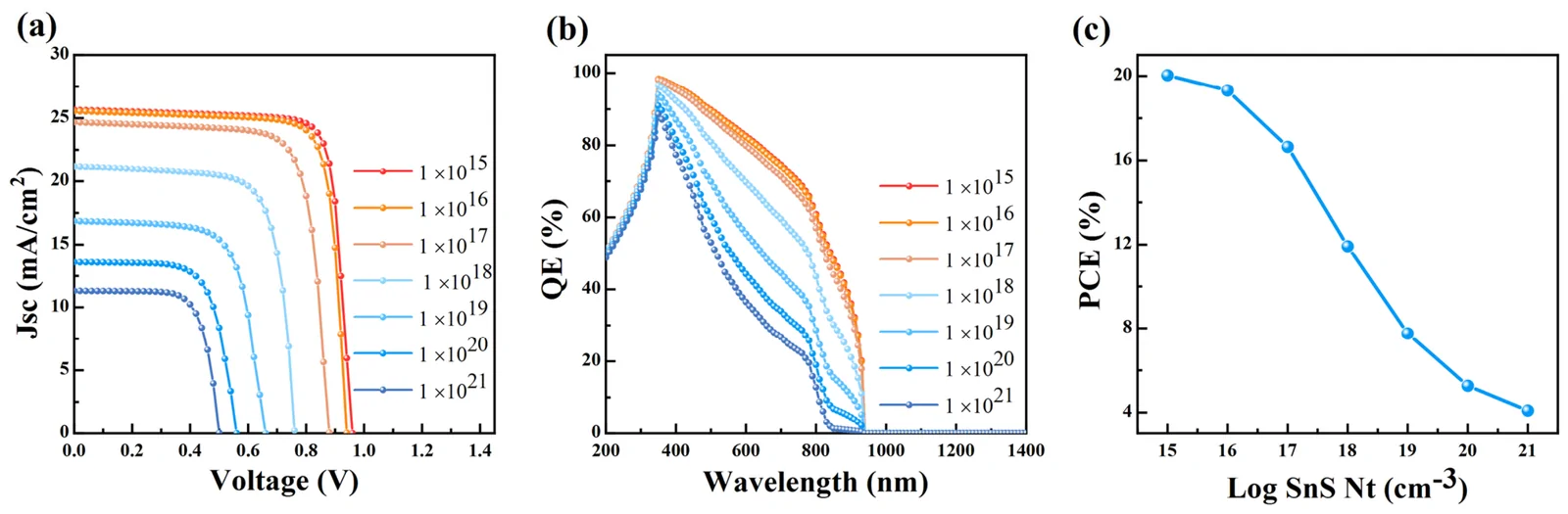
Influence of bulk defect density of SnS on solar cells. (**a**) *J-V* curves. (**b**) QE spectrum. (**c**) PCE.

As shown in Figure 8a, when the bulk defect density (N_t_) is below 10^16^ cm^−3^, the *J–V* curves exhibit typical high fill factor characteristics, with *J*_SC_ and *V*_OC_ stabilizing at approximately 25.56 mA/cm^2^ and 0.93 V, respectively. However, as N_t_ increases to the range of 10^17^ cm^−3^ to 10^21^ cm^−3^, both *J*_SC_ and *V*_OC_ reach notably lower values of 11.31 mA/cm^2^ and 0.50 V (N_t_ = 10^21^ cm^−3^), accompanied by a reduction in FF due to enhanced recombination. This phenomenon arises from high concentrations of bulk defects that serve as non-radiative recombination centers, which reduce the carrier lifetime (τ) and consequently limit quasi-Fermi level splitting, ultimately lowering charge extraction efficiency [54].

As illustrated in Figure 8b, the QE of the device exhibits stability for N_t_ below 10^16^ cm^−3^, in good agreement with the corresponding *J–V* characteristics. The QE maintains a strong spectral response across the visible wavelength range. On the other hand, a noticeable degradation in QE is observed with increasing N_t_. When N_t_ reaches 10^21^ cm^−3^, the QE at 700 nm drops from 74.59% to 26.76%. The underlying mechanism lies in bulk defects enhancing the bulk recombination probability of photogenerated carriers [54,59]. Particularly in absorption regions for long-wavelength photons, defect-induced recombination substantially impairs the carriers’ ability to diffuse to the electrodes. As shown in Figure 8c, when the value of N_t_ is below 10^15^ cm^−3^, the PCE remains largely stable at approximately 20.0%. PCE shows no significant fluctuation between 10^15^ and 10^16^ cm^−3^. However, when the N_t_ value is above 10^17^ cm^−3^, the PCE drops dramatically from 16.6% to 4.1%. The decline in performance stems from a mechanism involving multiple defects: an increase in Shockley–Read–Hall (SRH) recombination results in a reduced carrier lifetime, the presence of defect states generates energy barriers that impede charge transport, and a decrease in the SCR diminishes the built-in electric field that facilitates carrier separation. All of these synergistic effects reduce the efficiency of charge collection and enhance the intensity of non-radiative recombination. So we can conclude that bulk defects have a crucial impact on SnS TFSCs, and if the N_t_ of SnS films can be controlled below 10^16^ cm^−3^ during the actual production process, the PCE will remain at a high level.

### 3.5. Effect of the HTL Layers and Work Function of the Back Contact on the Device’s Performance

In solar cells, the work function (*Φ_m_*) of the back-contact metal represents the energy required for hole transfer from the semiconductor absorber layer to the electrode, determining how easily holes can be transferred. A low metal work function hinders hole transport, giving rise to partial hole recombination and reduced PCE [54]. Back-contact materials with work functions above 4.9 eV (e.g., Au, Pt) exhibit better device performance. Nevertheless, these metals are costly and not suited for large-scale production. To reduce manufacturing costs while maintaining performance, suitable HTLs are introduced to facilitate hole extraction. HTLs can modulate the energy barrier between the absorber and the back electrode, ensuring the formation of an optimal ohmic contact and thereby improving device performance. In this simulation study, the work function of the back-contact electrode was varied within the range of 4.4 eV to 5.2 eV to investigate the effects of various HTL materials (including NiO_x_, PEDOT:PSS, Spiro-OMeTAD, CuI, and CuSCN) on device performance [23,60,61,62,63]. Table 4 displays the simulated parameters for these HTL materials.

**Figure 9:**
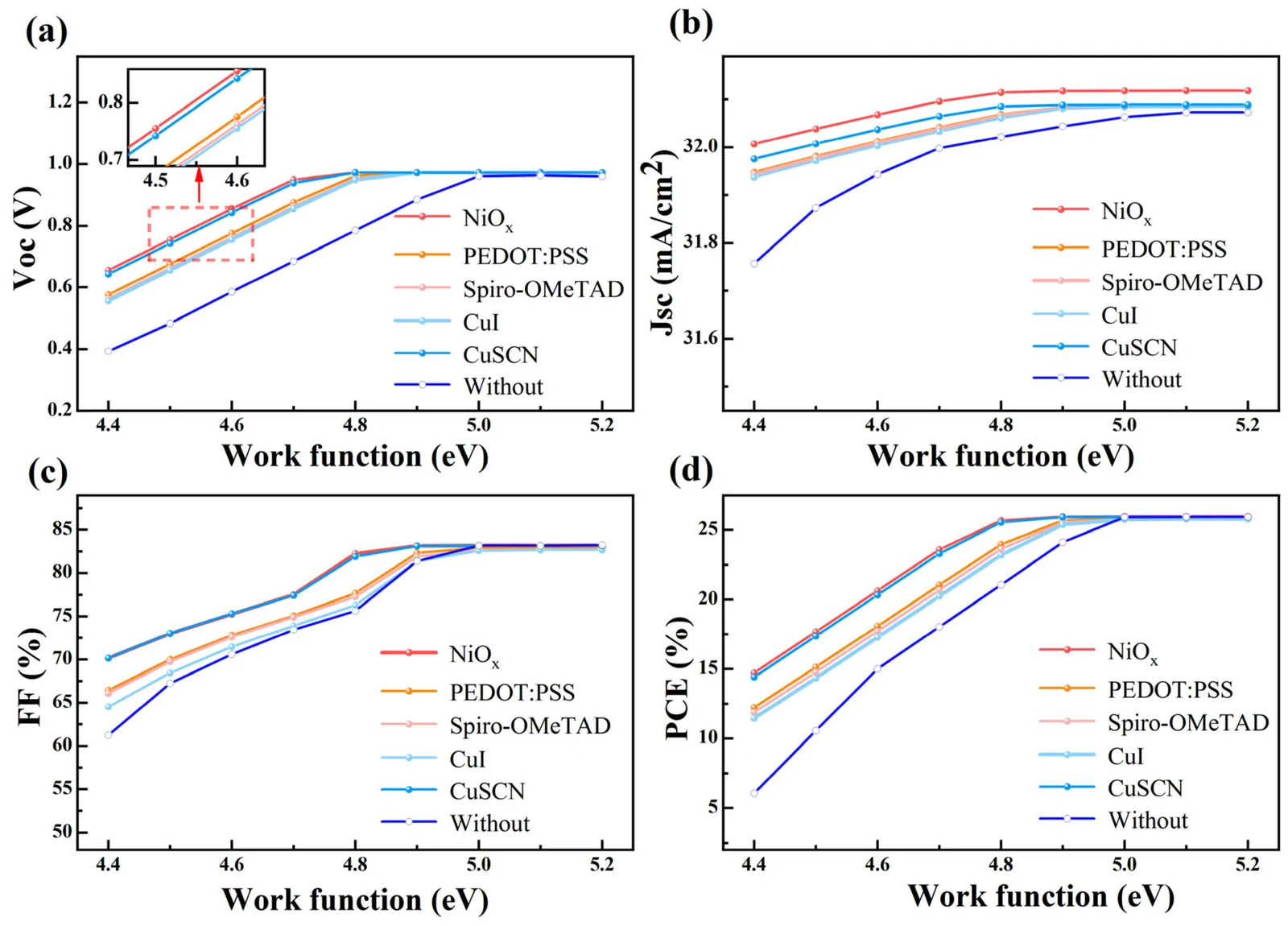
Variation of photovoltaic parameters with various HTLs attached to different back electron work functions. (**a**) *V*_OC_. (**b**) *J*_SC_. (**c**) FF. (**d**) PCE.

As can be seen from Figure 9, the addition of HTL layers greatly enhances several performance metrics of the solar cell. The *V*_OC_, *J*_SC_, FF, and PCE all show notable enhancements within the back electrode work function range of 4.4 eV to 4.9 eV. When the back electrode work function exceeds 4.9 eV, the photovoltaic parameters of the solar cell remain essentially stable. Compared to the device without an HTL layer, the NiO_x_ HTL demonstrates the most substantial performance improvement, followed by CuSCN, while CuI shows the least favorable enhancement. This is because NiO_x_ and CuSCN exhibit the best valence band alignment with the SnS absorber layer, effectively reducing the hole injection barrier and forming an efficient ohmic contact with minimal interfacial recombination losses. In contrast, the significant valence band mismatch between CuI and SnS creates a high energy barrier for hole extraction, leading to increased recombination rates. When NiO_x_ is employed as the HTL layer with a back electrode work function of 4.6 eV, the *V*_OC_, *J*_SC_, FF, and PCE improve from 0.59 V, 31.94 mA/cm^2^, 70.58%, and 15.01% (without HTL) to 0.86 V, 32.07 mA/cm^2^, 75.26%, and 20.64%, respectively. The efficiencies of cells with NiO_x_ as HTL reach their peak values of 25.96% at a back electrode work function above 4.9 eV.

### 3.6. Impact of Rs and Rsh on the Performance of the Device

The effects of series resistance (*R*_s_) and shunt resistance (*R*_sh_) on different aspects of the suggested PV device are studied in this section. In SnS-based solar cells, *R*_s_ serves as one of the critical factors limiting efficiency enhancement, primarily originating from intrinsic material resistance, interfacial resistance at the buffer layer and absorber layer interface, and non-ideal ohmic contacts formed by Schottky junctions (due to band misalignment) between the metal electrode and SnS [63,64].

**Figure 10:**
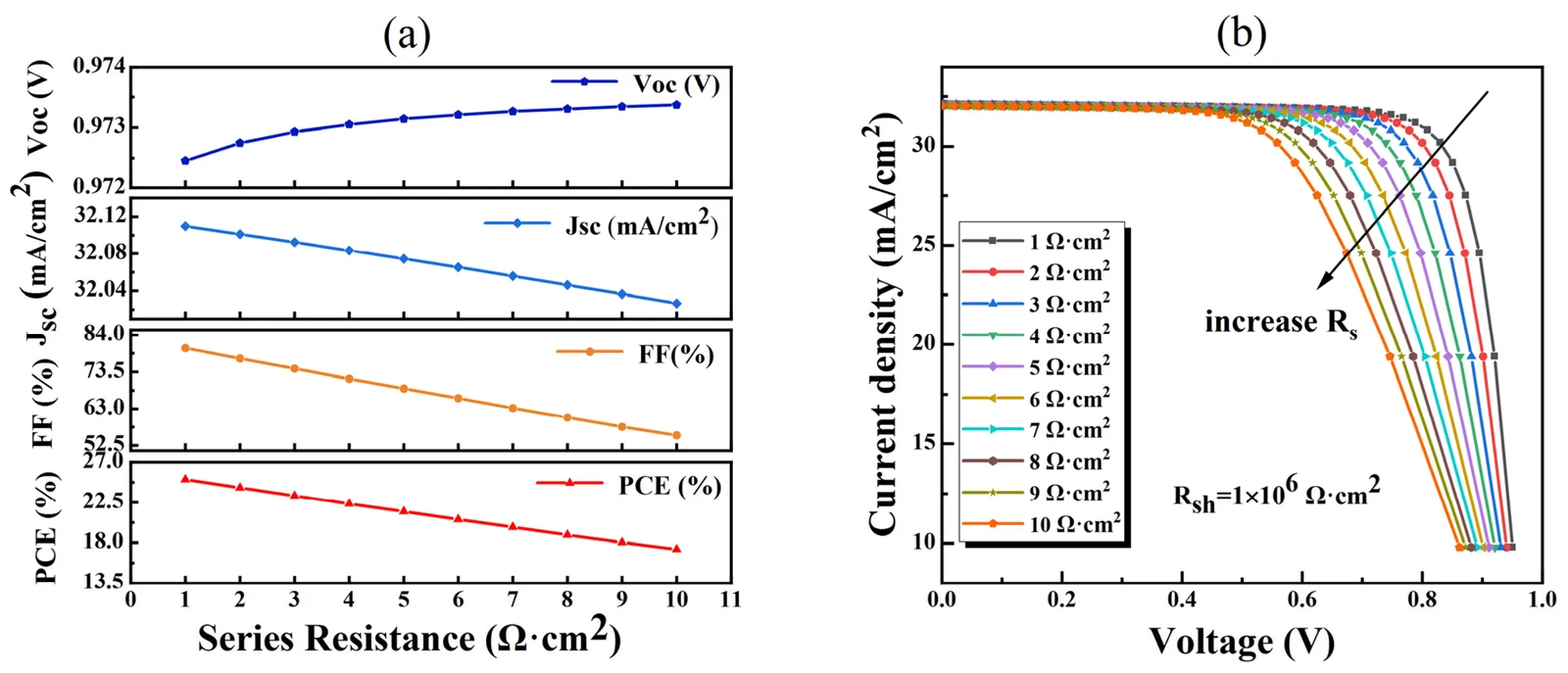
Impact of the series resistance on the performance of the device: (**a**) the output of JV parameters for the proposed devices. (**b**) The *J-V* characteristic curve.

With *R*_sh_ maintained constant at 10^6^ Ω·cm^2^, *R*_s_ was varied from 1 to 10 Ω·cm^2^. Figure 10 demonstrates how *R*_s_ affects the *J*–*V* parameter outputs and the *J–V* characteristic curves of the proposed device. As shown in Figure 10a, for the solar cells with the ITO/ZnS:In/SnS/NiO_x_/Au structure, as *R*_s_ increases from 1 to 10 Ω·cm^2^, *J*_SC_, FF, and PCE all exhibit a similar linearly decreasing trend, dropping from 32.11 mA/cm^2^, 80.23%, and 25.05% to 32.03 mA/cm^2^, 55.39%, and 17.27%, respectively. In contrast, the *V*_OC_ slightly increased from 0.9725 V to 0.9734 V. As observed from Figure 10b, the *J–V* characteristic curves for the proposed solar cell change shape with increasing Rs. These results indicate that elevated *R*_s_ leads to performance degradation and reduced PCE in the solar cell.

**Figure 11:**
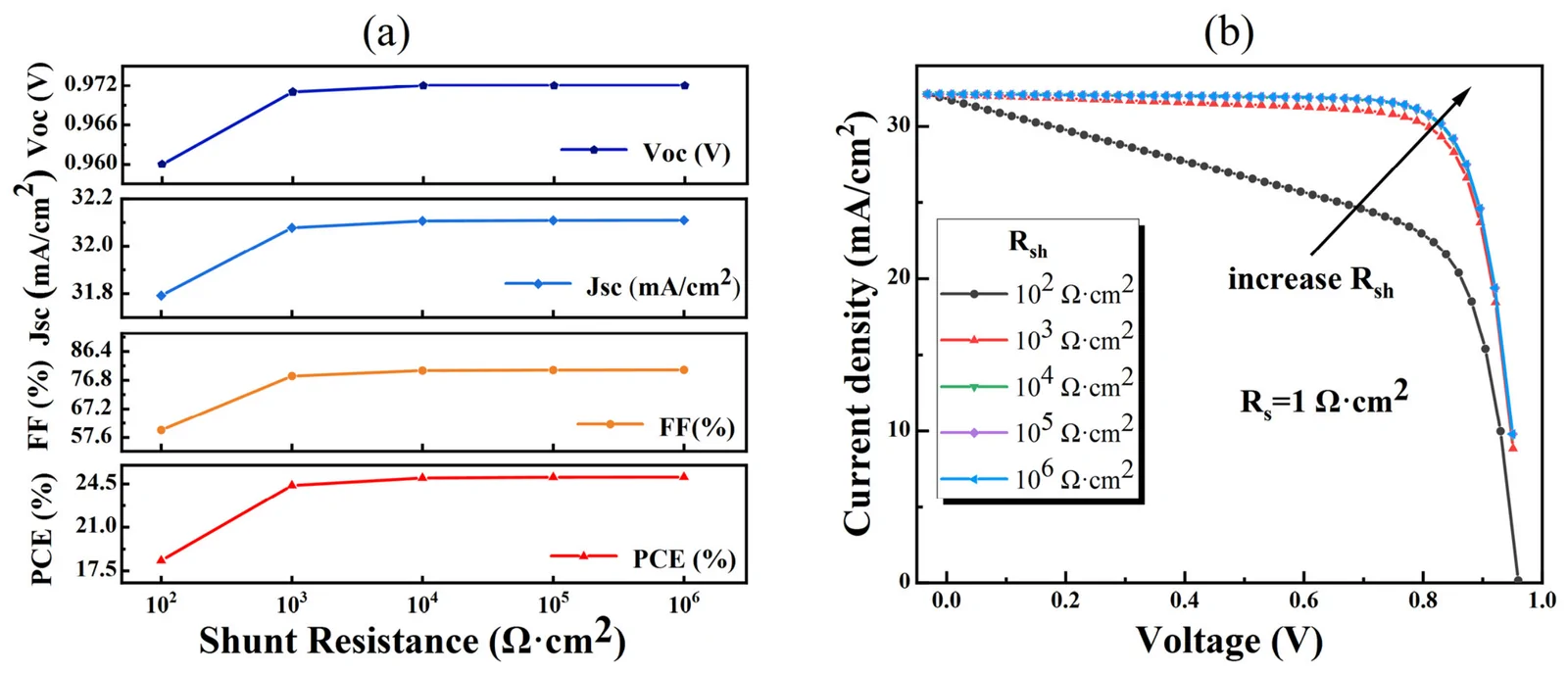
Impact of the shunt resistance on the performance of the device: (**a**) the output of IV parameters for the proposed devices. (**b**) The *J-V* characteristic curve.

In SnS solar cells, the shunt resistance primarily arises from bulk defect leakage, interfacial pinholes, grain boundary shunting, and edge shorting. *R*_sh_ reflects the leakage current pathways of the device, with lower values indicating more severe leakage. To investigate the impact of *R*_sh_ variations from 10^2^ to 10^6^ Ω·cm^2^ on device performance, we maintained *R*_s_ at a constant value of 1 Ω·cm^2^. Figure 11a and Figure 11b respectively illustrate the variations in photovoltaic output parameters and corresponding *J–V* characteristics of SnS-based solar cells with *R*_sh_. Simulation results reveal that when *R*_sh_ increases to 10^3^ Ω·cm^2^, all photovoltaic parameters exhibit significant improvements, and these parameters reach saturation when *R*_sh_ reaches 10^5^ Ω·cm^2^. As depicted in the simulation results of Figure 11b, a decrease in *R*_sh_ leads to degraded solar cell performance and reduced PCE. These results align with findings documented in the literature [64].

The above findings demonstrate that variations in the values of *R*_s_ and *R*_sh_ significantly impact solar cell performance, and these factors should be carefully considered during the design and optimization of solar cells. In order to improve the performance of solar cells, efforts should be made to minimize *R*_s_ to 1 Ω·cm^2^ while maximizing *R*_sh_ to be above 10^4^ Ω·cm^2^ [36,54].

### 3.7. Discussion on Theoretical Efficiency and Device Limitations

The power conversion efficiency of 27.01% is a theoretical limit obtained by multi-parameter global optimization of the cell structure under the SCAPS simulation framework. This result is based on a series of ideal material and interface assumptions and is not achievable under current experimental conditions.

#### 3.7.1. Justification of the 27.01% Theoretical Efficiency

The simulated PCE in our work is 27.01%, far below the theoretical Shockley–Queisser limit of about 32%, which is reasonable at the physical level. In addition, our simulation relies on several idealized assumptions: an ultra-low SnS bulk defect density to suppress SRH recombination. The favorable spike-type conduction band shift at the ZnS:In/SnS interface, the appropriate valence band arrangement at SnS/NiO_x_ for effective hole extraction and electron blocking, and the near-ideal ohmic contact with negligible potential barriers and series resistance. This does not consider secondary phase formation, grain boundary recombination, and interfacial diffusion. In addition, the one-dimensional SCAPS model eliminates the inherent film non-uniformity and leakage in real polycrystalline films.

#### 3.7.2. Dependence of Efficiency on Key Parameters

The device performance is dependent on several core parameters, including defect state density, heterojunction band shift, absorption layer doping concentration, thickness of each functional layer, and front and back-contact barriers.

Under initial conditions, the bulk defect density of the SnS absorber is set to 10^14^ cm^−3^, corresponding to ideal low-defect thin-film conditions. When the defect density rises to 10^17^ cm^−3^ (a typical value for traditional SnS thin films), PCE rapidly decreases to 4–5%, which is consistent with the experimental data summarized in Table 1. At higher defect concentrations, bulk SRH recombination becomes dominant, resulting in a significant decrease in open-circuit voltage. In terms of band alignment, the optimal efficiency was achieved under a small positive spike-type CBO. A large negative CBO will trigger severe interfacial tunneling recombination. The thickness of the absorber and p-type doping jointly balance the light absorption and carrier transport. Deviations from the optimal values will simultaneously reduce the open-circuit voltage and short-circuit current. In this simulation, an ideal ohmic contact was also assumed. In practical experiments, the contact barrier and series resistance directly degrade the fill factor.

#### 3.7.3. Difference Between Simulation and Experiment

It must be emphasized that the 27.01% PCE represents the theoretical limit obtained under ideal conditions, rather than the current experimental value. In practical experiments, secondary phases, grain boundary defects, interface diffusion, and non-ohmic contacts are easily formed during high-temperature annealing, which can affect the efficiency of the device.

#### 3.7.4. Practical Limitations of the Proposed Structure

Several inherent experimental bottlenecks hinder the practical realization of the proposed SnS-based device: (i) It remains challenging to stably suppress native intrinsic defects below 10^16^ cm^−3^. (ii) Heterojunction interfaces are susceptible to interdiffusion and oxidation, which deteriorate the designed band alignment. (iii) SnS possesses a narrow stable p-type doping window; heavy doping would introduce extra native defects. (iv) The fabrication of low-barrier, stable ohmic back contacts constitutes a bottleneck for SnS photovoltaic devices.

### 3.8. Summary of Final Optimized Device Parameters

To ensure the reproducibility and consistency of the simulation, all parameters of the final optimized ITO/ZnS:In/SnS/NiO_x_/Au/SLG device are summarized in Table 5.

The final optimized photovoltaic parameters of the device are: *V_OC_* = 1.002 V, *J_SC_* = 32.00 mA/cm^2^, FF = 84.2%, PCE = 27.01%. This optimal structure adopts eco-friendly ZnS:In as the electron transport layer and NiO_x_ as the hole transport layer, both of which are screened out from a series of candidate buffer and hole-transporting materials through systematic comparison. It is worth emphasizing that these photovoltaic metrics are limiting values under ideal simulation conditions. Practical devices inevitably show degraded performance due to poor thin-film quality and interfacial defect states. This set of optimized parameters can provide a theoretical basis for the subsequent experimental preparation of SnS heterojunction solar cells.

## 4. Conclusions

In this work, we employed the SCAPS-1D software to simulate and optimize the photovoltaic performance of SnS thin-film solar cells. According to the simulation results, SnS-based solar cells utilizing environmentally friendly and stable ZnSe and ZnS:In as buffer layers exhibited higher efficiencies compared to those using the traditional toxic CdS buffer layer. Moreover, the ZnS:In buffer layer demonstrated the best performance among the three. To achieve high-performance SnS-based solar cells, the thickness of the SnS absorber layer should be maintained at approximately 1.5 μm, with a doping concentration of about 10^18^ cm^−3^ and a bulk defect density below 10^16^ cm^−3^. The optimized thickness of the ZnS:In buffer layer is 0.08 μm, with a doping concentration of about 10^18^ cm^−3^. By selecting a metal with a work function higher than 4.9 eV (such as Au) as the back electrode and incorporating HTLs like NiO_x_, PEDOT:PSS, Spiro-OMeTAD, CuI, and CuSCN between the absorber layer and the back electrode, our comparative study and optimization revealed that NiO_x_ is the optimal HTL material for the envisioned solar cells in this research.

Furthermore, we investigated and optimized the electrical parameters of the proposed device by varying *R*_s_ and *R*_sh_. The final simulation results indicate that the optimized SnS solar cell achieves a *V*_OC_ of 1.002 V, a *J*_SC_ of 32.00 mA/cm^2^, FF of 84.2%, and PCE of 27.01%. It should be noted that this value corresponds to the optimal simulation results under idealized conditions. The actual device performance will be limited by the quality of the thin-film and interface defects. The aforementioned research findings suggest that the heterojunction solar cell with the structure of ITO/ZnS:In/SnS/NiO_x_/Au has the potential to become a high-efficiency, non-toxic, and low-cost solar cell. Moreover, the results of the simulation analysis provide a theoretical reference for the future design and fabrication of solar cells with similar structures.

## Figures and Tables

**Table 1:** The PV performance of SnS-based solar cells with various structures.

Type	Device Configurations	V_OC_ (V)	J_SC_ (mA/cm^2^)	FF (%)	PCE (%)
Experimental	Al/ITO/i-ZnO/CdS/SnS/Mo/SLG [26]	0.141	10.28	39.9	0.58
	p-SnS/n-CdS [4]	0.260	9.60	53.0	1.30
	SLG/Mo/SnS/CdS/i-ZnO/ZnO:B/Ni/Ag [27]	0.269	13.28	45.0	1.61
	Mo/SnS/ZnMgO/ZnO:Al/Ag [17]	0.575	9.96	36.4	2.10
	FTO/TiO_2_/SnS/P3HT/Ag [28]	0.850	7.35	45.0	2.81
	SLG/Mo/SnS/CdS/i-ZnO/AZO/Al [29]	0.334	18.90	55.5	3.50
	Si/SiO_2_/Mo/SnS/Zn(O,S):N/ZnO/ITO/Ag [18]	0.344	20.65	56.0	3.88
	SLG/Mo/SnS/CdS/i-ZnO/AZO/Al [30]	0.346	20.76	58.8	4.23
	ITO/ZnO/Zn(O,S):N/SnO_2_/SnS/Mo/glass [31]	0.372	20.20	58.0	4.36
	Mo/SnS/TiO_2_/FTO [19]	0.330	24.70	58.0	4.80
	TiO_2_/n-SnS/SnS/Ag/SnS/p-SnS/ITO [20]	0.450	17.13	68.0	5.24
Simulation	ITO/i-ZnO/Zn(O,S):N/SnO_2_/SnS [21]	0.410	27.99	67.9	7.68
	P+-SnS/SnS/CdS/ZnO [22]	0.730	17.00	65.0	8.15
	ZnS/SnS [32]	0.894	31.88	57.0	16.26
	ITO/CdS/SnS [24]	0.820	34.75	83.6	23.94
	ITO/CeO_2_/SnS/Spiro-OMeTAD/Mo [23]	0.887	25.65	85.6	25.65
	ITO/ZnS:In/SnS/NiO_x_/Au (This work)	1.002	32.00	84.2	27.01

**Table 2:** Simulation parameters of the proposed SnS-based solar cell [39,40,41,42,43].

Parameters	ITO [42,43]	Absorber SnS [25,44]	Buffer CdS [44]	Buffer ZnSe [45]	Buffer ZnS:In [46]
Thickness (nm)	500	1500	80	80	80
Band Gap (eV)	3.6	1.32	2.4	2.7	2.94–3.53
Electron Affinity(eV)	4.5	4.2	4.2	4.09	4.13
Dielectric Permittivity (relative)	8.9	14.0	10	10	8.2
CB effective density of states (cm^−3^)	2.2 × 10^18^	1.6 × 10^18^	2.2 × 10^18^	1.5 × 10^18^	6.4 × 10^18^
VB effective density of states (cm^−3^)	1.8 × 10^19^	8.3 × 10^18^	1.9 × 10^19^	1.8 × 10^19^	6.0 × 10^19^
Electron mobility (cm^2^/V·s)	10	20	100	50	250
Hole mobility (cm^2^/V·s)	10	100	25	20	40
Shallow uniform donor density (cm^−3^)	1.0 × 10^21^	0	1.0 × 10^18^	1.0 × 10^18^	1.0 × 10^18^
Shallow uniform acceptor density (cm^−3^)	0	5.0 × 10^16^	0	0	0
Defect type	SA	SD	SA	SA	SA
Energetic distribution	Gaussian	Gaussian	Gaussian	Gaussian	Gaussian
Defect density (cm^−3^)	1.0 × 10^10^	1.0 × 10^14^	1.0 × 10^15^	1.0 × 10^15^	1.0 × 10^15^

Note: CB = conduction band, VB = valence band, SA = single acceptor, and SD = single donor. The parameters listed are initial baseline values prior to optimization, taken from cited references. The final optimized parameters are summarized in Section 3.8.

**Table 3:** Interface parameters utilized in the ITO/CdS (ZnSe, ZnS:In)/SnS/Au/SLG device simulation.

Parameters	ZnSe/SnS Interface [41]	CdS/SnS Interface [25]	ZnS:In/SnS Interface [40]
Defect Type	Neutral	Neutral	Neutral
Capture Cross-Section of electrons and holes (cm^2^)	1.0 × 10^−19^	1.0 × 10^−19^	1.0 × 10^−19^
Energetic distribution	Single	Single	Single
Energy level (eV)	0.6	0.6	0.6
Total density N_t_ (cm^−3^)	1.0 × 10^11^	1.0 × 10^11^	1.0 × 10^11^

**Table 4:** Simulation parameters of HTLs.

Parameters	NiO_x_ [60]	PEDOT: PSS [61]	Spiro-OMeTAD [23]	CuI [62]	CuSCN [63]
Thickness (nm)	50	50	50	50	50
Band Gap (eV)	3.8	2.2	2.9	3.0	3.4
Electron Affinity(eV)	1.5	2.9	2.2	2.1	1.9
Dielectric Permittivity (relative)	10.7	3.0	3.0	6.5	10
CB effective density of states (cm^−3^)	2.8 × 10^19^	2.2 × 10^15^	2.2 × 10^18^	2.8 × 10^19^	1.7 × 10^19^
VB effective density of states (cm^−3^)	1.0 × 10^19^	1.8 × 10^19^	1.8 × 10^19^	1.0 × 10^19^	2.5 × 10^21^
Electron mobility (cm^2^/V·s)	100	2.0 × 10^−2^	2.0 × 10^−4^	1.7 × 10^−4^	1.0 × 10^−4^
Hole mobility (cm^2^/V·s)	25	2.0 × 10^−4^	2.0 × 10^−4^	1.7 × 10^−4^	1.0 × 10^−1^
Shallow uniform donor density (cm^−3^)	0	0	0	0	0
Shallow uniform acceptor density (cm^−3^)	1.0 × 10^17^	1.0 × 10^18^	2.0 × 10^18^	1.0 × 10^18^	1.0 × 10^18^

**Table 5:** Final optimized main parameters of the SnS-based heterojunction solar cell.

Layer	Thickness (μm)	Doping Concentration (cm^−3^)	Defect Density (cm^−3^)
ITO	0.5	1.0 × 10^21^	1.0 × 10^10^
ZnS:In (ETL)	0.08	1.0 × 10^18^	1.0 × 10^15^
SnS	1.5	1.0 × 10^18^	<1.0 × 10^16^
NiO_x_ (HTL)	0.05	1.0 × 10^17^	/

## Data Availability

Data are available upon request.

## References

[1] Patel M., Kim J., Kim Y.K. (2018). Growth of Large-Area SnS Films with Oriented 2D SnS Layers for Energy-Efficient Broadband Optoelectronics. Adv. Funct. Mater..

[2] Gedi S., Minnam Reddy V.R., Alhammadi S., Reddy Guddeti P., Kotte T.R.R., Park C., Kim W.K. (2019). Influence of deposition temperature on the efficiency of SnS solar cells. Sol. Energy.

[3] Rabkin A., Samuha S., Abutbul R.E., Ezersky V., Meshi L., Golan Y. (2015). New nanocrystalline materials: A pre-viously unknown simple cubic phase in the SnS binary system. Nano Lett..

[4] Ramakrishna Reddy K.T., Koteswara Reddy N., Miles R.W. (2006). Photovoltaic properties of SnS based solar cells. Sol. Energy Mater. Sol. Cells.

[5] Mahdi M.S., Ahmed N.M., Hmood A., Ibrahim K., Bououdina M. (2019). Comprehensive photoresponse study on high performance and flexible π-SnS photodetector with near-infrared response. Mater. Sci. Semicond. Process..

[6] Zheng D., Fang H., Long M., Wu F., Wang P., Gong F., Wu X., Ho J.C., Liao L., Hu W. (2018). High-Performance Near-Infrared Photodetectors Based on p-Type SnX (X = S, Se) Nanowires Grown via Chemical Vapor Deposition. ACS Nano.

[7] Burton L.A., Colombara D., Abellon R.D., Grozema F.C., Peter L.M., Savenije T.J., Dennler G., Walsh A. (2013). Synthesis, Characterization, and Electronic Structure of Single-Crystal SnS, Sn_2_S_3_, and SnS_2_. Chem. Mater..

[8] Arepalli V.K., Shin Y., Kim J. (2018). Influence of working pressure on the structural, optical, and electrical properties of RF-sputtered SnS thin films. Superlattices Microstruct..

[9] Ham G., Shin S., Park J., Choi H., Kim J., Lee Y.-A., Seo H., Jeon H. (2013). Tuning the Electronic Structure of Tin Sulfides Grown by Atomic Layer Deposition. ACS Appl. Mater. Interfaces.

[10] Cho J.Y., Sinha S., Gang M.G., Heo J. (2019). Controlled thickness of a chemical-bath-deposited CdS buffer layer for a SnS thin film solar cell with more than 3% efficiency. J. Alloys. Compd..

[11] Javed A., Khan N., Bashir S., Ahmad M., Bashir M. (2020). Thickness dependent structural, electrical and optical properties of cubic SnS thin films. Mater. Chem. Phys..

[12] Gedi S., Reddy V.R.M., Kang J., Jeon C.-W. (2017). Impact of high temperature and short period annealing on SnS films deposited by E-beam evaporation. Appl. Surf. Sci..

[13] Voznyi A., Kosyak V., Yeromenko Y., Keller J., Bērziņa A., Shamardin A., Iatsunskyi I., Shpetnyi I., Plotnikov S., Opanasyuk A. (2020). Close-spaced sublimation of SnS absorber layers and SnS/CdS heterojunction solar cells with Mo and Ti back metal contacts. Thin Solid Films.

[14] Gunasekaran M., Ichimura M. (2007). Photovoltaic cells based on pulsed electrochemically deposited SnS and photochemically deposited CdS and Cd1−*x*Zn*x*S. Sol. Energy Mater. Sol. Cells.

[15] Ding D., Rath T., Lanzetta L., Manuel Marin-Beloqui J., Haque S.A. (2018). Efficient Hybrid Solar Cells Based on Solution Processed Mesoporous TiO_2_/Tin(II) Sulfide Heterojunctions. ACS Appl. Energy Mater..

[16] Ur Rehman U., Almufarij R.S., Shokralla E.A., Sahar K.U., Abd-Elwahed A.R., Ragab I., Fahmy M.A., Alrefaee S.H., Ashfaq A., Wang C.-M. (2025). Numerical design and optimization of Sb_2_S_3_/SnS_2_ heterojunction solar cells: Performance analysis and insights. Mater. Sci. Eng. B.

[17] Takano Y., Oyaizu K. (2018). Fabrication of SnS-MgSnO heterojunction solar cells using vacuum thermal evaporation and sol-gel method. Mater. Lett..

[18] Steinmann V., Jaramillo R., Hartman K., Chakraborty R., Brandt R.E., Poindexter J.R., Lee Y.S., Sun L., Polizzotti A., Park H.H. (2014). 3.88% Efficient Tin Sulfide Solar Cells using Congruent Thermal Evaporation. Adv. Mater..

[19] Yun H.-S., Park B., Choi Y.C., Im J., Shin T.J., Seok S.I. (2019). Efficient Nanostructured TiO_2_/SnS Heterojunction Solar Cells. Adv. Energy Mater..

[20] Nguyen T.D., Dang V.T., Hung N.M., Arepalli V.K., Kim J., Raj M., Nguyen T.T.O. (2021). Synthesis of Ag-embedded SnS films by the RF method for photovoltaic applications. Surf. Interfaces.

[21] Minbashi M., Ghobadi A., Ehsani M.H., Rezagholipour Dizaji H., Memarian N. (2018). Simulation of high efficiency SnS-based solar cells with SCAPS. Sol. Energy.

[22] Kumar A., Prabu R.T., Das A. (2022). Configuration analysis of SnS based solar cells for high-efficiency devices. Opt. Quant. Electron..

[23] Tiwari P., Alotaibi M.F., Al-Hadeethi Y., Srivastava V., Arkook B., Sadanand, Lohia P., Dwivedi D.K., Umar A., Algadi H. (2022). Design and Simulation of Efficient SnS-Based Solar Cell Using Spiro-OMeTAD as Hole Transport Layer. Nanomaterials.

[24] Oublal E., Sahal M., Abdelkadir A.A. (2022). New theoretical analysis of a novel hetero-junction SnS/CdS solar cell with homo-junction P–P+ in the rear face-numerical approach. Curr. Appl. Phys..

[25] Hosen A., Ahmed S.R.A. (2022). Performance analysis of SnS solar cell with a hole transport layer based on experimentally extracted device parameters. J. Alloys. Compd..

[26] Jaramillo R., Steinmann V., Yang C., Hartman K., Chakraborty R., Poindexter J.R., Castillo M.L., Gordon R., Buonassisi T. (2015). Making Record-efficiency SnS Solar Cells by Thermal Evaporation and Atomic Layer Deposition. J. Vis. Exp..

[27] Kang J., Kwon S.-M., Yang S.H., Cha J.-H., Bae J.A., Jeon C.-W. (2017). Control of the microstructure of SnS photovoltaic absorber using a seed layer and its impact on the solar cell performance. J. Alloys. Compd..

[28] Zafar A., Ahmad K.S., Jaffri S.B., Sohail M. (2020). Physical vapor deposition of SnS:PbS-dithiocarbamate chalcogenide semiconductor thin films: Elucidation of optoelectronic and electrochemical features. Phosphorus Sulfur Silicon Relat. Elem..

[29] Yadav R.K., Pawar P.S., Neerugatti K.E., Nandi R., Cho J.Y., Heo J. (2021). Effect of intrinsic ZnO thickness on the performance of SnS/CdS-based thin-film solar cells. Curr. Appl. Phys..

[30] Cho J.Y., Kim S., Nandi R., Jang J., Yun H.-S., Enkhbayar E., Kim J.H., Lee D.-K., Chung C.-H., Kim J. (2020). Achieving over 4% efficiency for SnS/CdS thin-film solar cells by improving the heterojunction interface quality. J. Mater. Chem. A.

[31] Sinsermsuksakul P., Sun L., Lee S.W., Park H.H., Kim S.B., Yang C., Gordon R.G. (2014). Overcoming Efficiency Limitations of SnS-Based Solar Cells. Adv. Energy Mater..

[32] Xu J., Yang Y. (2014). Study on the performances of SnS heterojunctions by numerical analysis. Energy Convers. Manag..

[33] Andrade-Arvizu J.A., Courel-Piedrahita M., Vigil-Galán O. (2015). SnS-based thin film solar cells: Perspectives over the last 25 years. J. Mater. Sci. Mater. Electron..

[34] Rühle S. (2016). Tabulated values of the Shockley–Queisser limit for single junction solar cells. Sol. Energy.

[35] Lindwall G., Shang S., Kelly N.R., Anderson T., Liu Z.-K. (2016). Thermodynamics of the S–Sn system: Implication for synthesis of earth abundant photovoltaic absorber materials. Sol. Energy.

[36] Abbas K.J., Bahrami A. (2024). Investigating the potential of WO_3_ and WS_2_ as Cd-free buffer layers in Sb_2_Se_3_-Based thin-film solar cells: A numerical study with SCAPS-1D software. Sol. Energy Mater. Sol. Cells.

[37] Movla H. (2014). Optimization of the CIGS based thin film solar cells: Numerical simulation and analysis. Optik.

[38] Al Mahmud J., Rahman F., Kuddus A., Ali H., Islam A.T.M.S., Haque D., Al Ahmed S.R., Mushtaq M., Ismail A.B.M. (2023). Design and analysis of a SnS_2_/WS_2_/V_2_O_5_ double-heterojunction toward high-performance photovoltaics. Energy Adv..

[39] Huang Z.-P., Chen Y.-X., Huang Z.-H., Lin W.-W., Mao Y., Lin L.-M., Yao L.-Q., Li H., Cai L.-P., Chen G.-L. (2023). Simulation of highly efficient GeSe-based solar cells with SCAPS-1D. Heliyon.

[40] Abdallah B., Alnama K., Nasrallah F. (2019). Deposition of ZnS thin films by electron beam evaporation technique, effect of thickness on the crystallographic and optical properties. Mod. Phys. Lett. B.

[41] Ben Hjal A., Yazdanpanah A., Colusso E., Tormena N., Nicola D.D., Dolcet P., Gross S., Trivellin N., Alouani K., Brunelli K. (2025). Enhancing kesterite-based thin-film solar cells: A dual-strategy approach utilizing SnS back surface field and eco-friendly ZnSe electron transport layer. Appl. Surf. Sci..

[42] Huang X., Zhang Y., Fang L., Zhou X., Yu Q., Meng K. (2024). Analysis and design of 25.3% efficient Sb_2_Se_3_ solar cells by numerical simulation. Results Opt..

[43] Ait Abdelkadir A., Sahal M., Oublal E., Kumar N., Benami A. (2023). New Sb_2_Se_3_-based solar cell for achieving high efficiency theoretical modeling. Opt. Quant. Electron..

[44] Islam B., Hosen A., Khan T.M., Rahman M.F., Rahman M.H., Islam M.S., Ahmed S.R.A. (2024). Simulating the Effect of Inserting Sb_2_S_3_ as Hole Transport Layer on SnS-Based Thin-Film Solar Cells. J. Electron. Mater..

[45] Sharma R., Himanshu, Patel S.L., Chander S., Kannan M.D., Dhaka M.S. (2020). Physical properties of ZnSe thin films: Air and vacuum annealing evolution to buffer layer applications. Phys. Lett. A.

[46] Rahman M.F., Hasan M.K., Chowdhury M., Islam M.R., Rahman M.H., Rahman M.A., Al Ahmed S.R., Ismail A.B.M., Amami M., Hossain M.K. (2023). A qualitative Design and optimization of CIGS-based Solar Cells with Sn2S3 Back Surface Field: A plan for achieving 21.83% efficiency. Heliyon.

[47] Sobayel K., Shahinuzzaman M., Amin N., Karim M.R., Dar M.A., Gul R., Alghoul M.A., Sopian K., Hasan A.K.M., Akhtaruzzaman M. (2020). Efficiency enhancement of CIGS solar cell by WS_2_ as window layer through numerical modelling tool. Sol. Energy.

[48] Mamta, Maurya K.K., Singh V.N. (2021). Sb_2_Se_3_ versus Sb_2_S_3_ solar cell: A numerical simulation. Sol. Energy.

[49] Alsulami A. (2025). Exploration of the impact of indium-doping on the microstructural, dispersion, linear, and nonlinear optical properties of the ZnS thin films. Phys. B Condens. Matter.

[50] Rahman M.F., Monnaf M.A., Amami M., Ben Farhat L., Rahman M.A. (2024). Improving the efficiency above 35% of MoS_2_-based solar cells by through optimization of various wide-bandgap *S*-chalcogenides ETL. J. Phys. Chem. Solids.

[51] Kumari R., Kumar R., Singh V. (2022). SnTe as a BSF enhances the performance of Sb_2_Se_3_ based solar cell: A numerical approach. Heliyon.

[52] Fentahun D.A., Tyagi A., Kar K.K. (2021). Numerically investigating the AZO/Cu_2_O heterojunction solar cell using ZnO/CdS buffer layer. Optik.

[53] Cheragee S.H., Alam M.J. (2023). Device modeling and numerical study of a double absorber solar cell using a variety of electron transport materials. Heliyon.

[54] Ahmed S.R.A., Sunny A., Rahman S. (2021). Performance enhancement of Sb_2_Se_3_ solar cell using a back surface field layer: A numerical simulation approach. Sol. Energy Mater. Sol. Cells.

[55] Rai N., Rai S., Singh P.K., Lohia P., Dwivedi D.K. (2020). Analysis of various ETL materials for an efficient perovskite solar cell by numerical simulation. J. Mater. Sci. Mater. Electron..

[56] Aliaghayee M. (2023). Optimization of the Perovskite Solar Cell Design with Layer Thickness Engineering for Improving the Photovoltaic Response Using SCAPS-1D. J. Electron. Mater..

[57] Jafarzadeh F., Aghili H., Nikbakht H., Javadpour S. (2022). Design and optimization of highly efficient perovskite/homojunction SnS tandem solar cells using SCAPS-1D. Sol. Energy.

[58] Ablekim T., Colegrove E., Metzger W.K. (2018). Interface Engineering for 25% CdTe Solar Cells. ACS Appl. Energy Mater..

[59] Atowar Rahman M. (2021). Enhancing the photovoltaic performance of Cd-free Cu_2_ZnSnS_4_ heterojunction solar cells using SnS HTL and TiO_2_ ETL. Sol. Energy.

[60] Ahmmed S., Aktar A., Hossain J., Ismail A.B.M. (2020). Enhancing the open circuit voltage of the SnS based heterojunction solar cell using NiO HTL. Sol. Energy.

[61] Ezealigo B.N., Nwanya A.C., Simo A., Osuji R.U., Bucher R., Maaza M., Ezema F.I. (2019). Optical and electrochemical capacitive properties of copper (I) iodide thin film deposited by SILAR method. Arab. J. Chem..

[62] Liu J., Zhang Y., Liu C., Peng M., Yu A., Kou J., Liu W., Zhai J., Liu J. (2016). Piezo-phototronic effect enhanced UV photodetector based on CuI/ZnO double-shell grown on flexible copper microwire. Nanoscale Res. Lett..

[63] Li Z.-Q., Ni M., Feng X.-D. (2020). Simulation of the Sb_2_Se_3_ solar cell with a hole transport layer. Mater. Res. Express.

[64] Kafashan H., Bahrami A. (2024). CIGS solar cells using ZrS_2_ as buffer layer: Numerical simulation. Optik.

